# Acute Myeloid Leukemia Post Cytotoxic Therapy in Breast Cancer Survivors—Over 23 Years of Single Center Analysis

**DOI:** 10.3390/jcm13040989

**Published:** 2024-02-08

**Authors:** Monika Adamska, Ewelina Kowal-Wiśniewska, Marta Barańska, Anna Przybyłowicz-Chalecka, Anna Łojko-Dankowska, Monika Joks, Małgorzata Jarmuż-Szymczak, Lidia Gil

**Affiliations:** 1 Department of Hematology and Bone Marrow Transplantation, Poznan University of Medical Sciences, 60-569 Poznan, Poland; 2 Doctoral School, Poznan University of Medical Sciences, 60-812 Poznan, Poland; 3 Institute of Human Genetics, Polish Academy of Sciences, 60-479 Poznan, Poland

**Keywords:** breast cancer, acute myeloid leukemia post cytotoxic therapy, allogenic hematopoietic cell transplantation, prior cytotoxic therapy, inherited cancer susceptibility

## Abstract

**Background**: Acute myeloid leukemia post cytotoxic therapy (AML-pCT) among breast cancer (BC) survivors represents a life-threatening complication. This study aims to assess the clinical outcomes of AML-pCT post BC. **Methods**: An analysis of all AML patients treated at a single hematology center (2000–2023) was performed to select patients with AML-pCT post BC. We applied the 2022 ELN criteria to define the genetic risk. **Results**: Among 847 AML patients, 28 were diagnosed with AML-pCT following BC. Complex karyotype (CK) occurred in 23.8% of patients. The median overall survival (OS) was 40 months. The survival outcomes were better after allogenic hematopoietic stem cell transplantation (alloHCT) treatment compared to chemotherapy alone (median OS: 47 versus 7 months, *p* = 0.008). Patients demonstrating CK showed lower survival compared to those without CK (2-year OS: 25.0% versus 66.2%, *p* = 0.0048). The multivariable Cox proportional hazards regression model indicated that treatment with alloHCT emerged as a significant factor associated with improved OS. The treatment was associated with superior OS (HR = 0.07, 95% CI = 0.01–0.86, *p* = 0.04). **Conclusions**: Patients with AML-pCT following BC were characterized with the highest frequency of adverse genetic risk profiles and demonstrated worse survival rates. AlloHCT should be performed as early as possible in such patients. The growing need for studies on inherited cancer susceptibility underscores the importance of close AML-pCT development monitoring in BC survivors.

## 1. Introduction

Breast cancer (BC) represents one of the most frequent malignancies, with a prevalence of 9.1 million cases worldwide in 2020 [1]. Although uncommon, one of the most serious, long-term complications after cytotoxic therapy for the treatment of BC is the development of acute myeloid leukemia post cytotoxic therapy (AML-pCT). 

In the WHO 2022 classification of myeloid neoplasms, AML-pCT is recognized as a distinct acute myeloid leukemia (AML) subtype, representing 10–20% of all AML cases [2]. Prior exposure to DNA-damaging agents, commonly used for the adjuvant and neo-adjuvant treatment of BC, triggers the leukemogenesis and increases the risk of AML-pCT. Furthermore, the development of AML-pCT in patients previously affected by BC may also be associated with inherited cancer susceptibility [3,4]. Among patients with AML-pCT following BC, germline mutations were detected in 21%, with the highest frequency observed in the *BRCA1*, *TP53,* and *BRCA2* genes [5]. Myeloid neoplasms are diagnosed in approximately 0.6–1.8% of patients following BC treatment, typically manifesting 5–10 years following BC diagnosis [6,7]. The development of AML-pCT is associated with poor outcomes and a higher incidence of adverse molecular findings, such as the presence of a *TP53* gene mutation and complex karyotype (CK) [8]. Different factors may lead to an adverse prognosis in AML-pCT patients, including side effects from prior cancer cytotoxic therapy, comorbidities, and age [9]. Irrespective of the promising results of novel agents in AML-pCT, such as CPX-531 or venetoclax, allogenic hematopoietic cell transplantation (alloHCT) represents the only curative treatment option [10,11]. Importantly, a prior history of cancer serves as a negative non-relapse mortality prognostic factor, incorporated into the HCT-CI score [12]. However, the participation of AML-pCT patients in clinical trials involving novel agents is limited.

With the advances in the treatment of BC (5-year OS: 66–99%), the global rise in BC survivors is evident and, as a consequence, the number of AML-pCT cases is expected to grow.

In contrast to our previous report on AML-pCT, in this retrospective single center study, we focus only on AML-pCT patients previously affected by BC, recognized as one of the most frequent solid tumors (ST) worldwide [13]. Through an extensive analysis of treatment complications and genetic features, we have focused on describing the clinical characteristics and treatment outcomes of patients with AML-pCT and preceding BC over the past 23 years. 

## 2. Materials and Methods

We have performed a retrospective analysis of the medical records of all 847 AML patients from a single hematology center (Poznan, Poland). Our selection criteria included subjects with the following characteristics: (1) a diagnosis of AML-pCT with previous BC according to the 2022 World Health Organization (WHO) criteria, (2) age ≥ 18 years, and (3) hospitalization between January 2000 and March 2023. In our previous report, AML-pCT following BC was the predominant primary malignancy [13]. Thus, we decided to expand the study group and conduct a separate analysis. The clinical parameters examined in this group were identical to those in our previous study [13] and comprised factors such as age, race, cytogenetic and molecular data (performed at the time of AML-pCT diagnosis according established guidelines), laboratory parameters, treatment type, treatment toxicity, infections after treatment, graft versus host disease (GvHD), response to treatment, and BC medical data (date of diagnosis, latency period, type of cytotoxic therapy). The 2022 European Leukemia Net (ELN) criteria were applied to define the genetic risk associated with AML-pCT [14]. As the cytotoxic therapy for BC varied among different Polish oncological centers between 1982 and 2019, and due to the high mortality of AML-pCT in post BC patients, we collected limited data concerning BC treatment and genetics.

This study was approved by the bioethical committee of Poznan University of Medical Sciences (approval no.1040/19) and designed according to the Declaration of Helsinki. 

### Statistical Analysis

Statistical analyses were performed with the use of MedCalc Statistical Software Ltd, Ostend, Belgium (v. 19.5.3). The normality of data distribution was assessed via the Shapiro–Wilk test. Statistical significance was determined at *p* < 0.05. The following statistical tests were used for comparisons: the Mann–Whitney test (for the quantitative variables without normal distribution), Student’s *t*-test (for the quantitative variables with a normal distribution) and Pearson’s chi-squared test (for the qualitative variables). Progression-free survival (PFS) and overall survival (OS) represented the primary study endpoints. OS was defined as the interval between AML-pCT diagnosis and the last contact or death (for individuals undergoing alloHCT, this interval was calculated between the procedure and the last contact or death). PFS was established as the interval between diagnosis and death or relapse (for individuals undergoing alloHCT, it was calculated between the procedure and death or relapse). The Kaplan–Meier method (as compared with the use of the log-rank test), was used to calculate OS and PFS. The Cox proportional-hazards regression model was employed to analyse the prognostic factors. In the multivariable analyses, (hazard ratios; 95% confidence intervals) we included selected factors. 

## 3. Results

### 3.1. AML-pCT after Breast Cancer, Clinical Characteristics

Out of the 847 AML patients included in this study, 80 individuals (9.4%) were diagnosed with AML-pCT. STs preceded AML-pCT in 70.0% of these cases, with BC representing the most prevalent tumor subtype, accounting for 50.0% of all STs. AML-pCT after prior cytotoxic BC therapy occurred in 28 (34.6%) patients, with a median age of 57.5 (50.5–64.5) years. One patient underwent cytotoxic therapy for both BC (radiotherapy and chemotherapy) and uterine cancer (radiotherapy and chemotherapy), and one patient received cytotoxic therapy for two different BSs (Table 1). One patient was a carrier of both *PALB2* and *CHEK2* germline mutations and underwent prophylactic hysterectomy and prophylactic mastectomy. In one BC patient, a *CHEK2* mutation was detected. Details regarding cytotoxic therapy for BC and BC genetics are listed in Table 1.

Most of the patients with AML-pCT preceded by BC (75.0%) were diagnosed after 2015. The median latency time was 5.0 (4.0–7.0) years. In the majority of BC survivors (12/28; 42.9%) AML-pCT was observed to be triggered by both chemotherapy and radiotherapy, in 28.6% by radiotherapy only, and in 25.0% by chemotherapy alone. Additionally, 57.1% of patients received hormone therapy and 100.0% of patients underwent surgical interventions. The median latency times were comparable among different anticancer treatments: 5.5 years for radiotherapy, 5.0 years for chemotherapy, or 5.0 years for both (*p* = 0.96). Among AML-pCT cases with preceding BC, 21.4% (six out of 28) were categorized as AML-pCT myelodysplasia-related (AML-pCT-MR) after BC, with five patients developing myelodysplastic neoplasms post cytotoxic therapy (MDS-pCT) and one patient showing AML-pCT-MR post BC based on cytogenetic abnormalities. In the presented group, one patient suffered from acute promyelocytic leukemia post cytotoxic therapy (APL-pCT) following BC. (Table 2).

### 3.2. Molecular and Cytogenetic Characteristics of AML-pCT after Breast Cancer

Cytogenetic abnormalities were noted in 90.5% of patients, with the most frequently being the 17p13 deletion and CK, occurring at 23.8% and 19.0%, respectively. These findings are comparable to those in our previous report, which encompassed the general AML-pCT population [13]. Additionally, *FLT3-ITD* and *NPM1* mutations were detected in 17.6% and 14.3% of individuals, respectively.

In terms of the 2022 ELN genetic risk category, the majority of AML-pCT patients with preceding BC patients were classified as adverse (54.2%), with 33.4% classified as intermediate, and 12.5% as favorable (Table 3).

Among AML-pCT-MR patients after BC (*n* = 6), 80.0% of individuals were identified as having an adverse prognosis, and 20.0% were categorized as intermediate risk (2022 ELN criteria). The 17p13 deletion was detected in 25.0% (one-fourth) of the AML-pCT-MR patients post BC, while *FLT3-ITD* presence was detected in 33.3% (one-third) of the cases.

### 3.3. Treatment of AML-pCT after Breast Cancer Cytotoxic Therapy

Intensive treatment (77.8%), which included induction therapy (cytarabine and daunorubicin [*n* = 16], eventually supplemented with cladribine [*n* = 3]), consolidation therapy (high dose cytarabine), and eventually alloHCT, was applied only to medically fit patients. This treatment strategy mirrors that applied to the general AML-pCT population in our previous report [13]. Only two AML-pCT after BC *FLT3*-positive patients received molecular targeted therapy with midostaurin. Treatment of APL-pCT after BC included arsenic trioxide and all-trans retinoic acid with idarubicine and mitoxantrone (one patient). A proportion of 17.9% of patients (5/28) underwent primary non-intensive treatment, which encompassed azacitidine (*n* = 2) or venetoclax with azacitidine (*n* = 3). A proportion of 10.7% (3/28) of patients underwent palliative care (6-mercaptopurine or metothrexate, hydroxyurea, and supportive care) (Table 4). The median age of individuals undergoing non-intensive treatment and intensive treatment was 70.0 and 55.0 years, respectively (*p* = 0.001).

A proportion of 48.0% (12/25) of AML-pCT patients with preceding BC underwent alloHCT in the course of treatment. These patients had a median age of 55.0 (IQR: 46.5–58.0) years. Stem cells were sourced from peripheral blood (100.0%). In 75.0% of individuals, alloHCT was performed in the first complete remission (CR1), and in 25.0% in CR2, CR3, or active disease. Myeloablative conditioning (MAC) was performed in 25.0%, and reduced-intensity conditioning was used in 75.0% of AML-pCT after BC patients undergoing alloHCT (Table 4). A proportion of 50.0% of AML-pCT patients with preceding BC that received alloHCT were classified as intermediate risk, 20.0% as favorable risk, and 30.0% as adverse risk (2022 ELN criteria).

### 3.4. AML-pCT after Breast Cancer Survival

**Figure 1:**
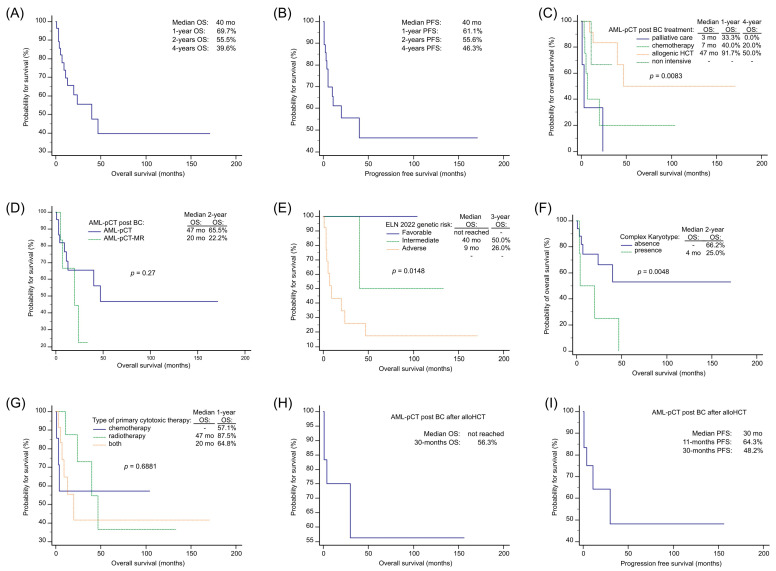
Overall survival and progression-free survival in AML-pCT after BC patients. Legend: alloHCT: allogenic hematopoietic cell transplantation, AML-pCT: acute myeloid leukemia post cytotoxic therapy, AML-pCT-MR: AML-pCT myelodysplasia-related, BC: breast cancer, mo: months, OS: overall survival, PFS: progression-free survival.

With a median follow-up of 19 months, the overall median OS and PFS for AML-pCT patients with preceding BC was 40 months (Figure 1A,B). Survival outcomes demonstrated significant differences based on the treatment approach undertaken (*p* = 0.0083), with a median OS of 7 months among patients undergoing intensive treatment (chemotherapy only), 47 months for those treated with alloHCT, and 3 months for those subjected to palliative care. The OS was not determined for those undergoing non-intensive treatment (Figure 1C). OS was shorter for AML-pCT-MR patients compared to AML-pCT patients following BC (2-years OS: 65.5% and 22.2%; *p* = 0.269) (Figure 1D). Survival rates differed among the 2022 ELN genetic risk subgroups (*p* = 0.0148), with a median OS of 40.0 months for the intermediate, 9.0 months for the adverse risk subgroup, and an unreached median for the favorable subgroup (Figure 1E). AML-pCT patients after BC with a complex karyotype (CK) demonstrated inferior survival outcomes (*p* = 0.0048) (Figure 1F).

One patient with APL-pCT post BC remained in CR1 after treatment, with an overall survival of 81 months.

Regarding previous oncological cytotoxic therapy, survival rates did not differ among patients that received chemotherapy, radiotherapy, or a combination of both (*p* = 0.6881) with 1-year OS 57.1%, 87.5%, and 64.8% (Figure 1G). Among AML-pCT after BC individuals undergoing alloHCT 30-month OS and PFS were 56.3% and 48.2%, respectively (Figure 1H,I). The median follow-up for AML-pCT after BC alloHCT recipients was 26.0 months.

In both univariable and multivariate Cox proportional hazards regression models, treatment with alloHCT emerged as a significant factor associated with improved OS (all analyzed factors are listed in Table 5). In the univariate Cox proportional hazards regression model, AML-pCT-MR post BC subtype among patients undergoing intensive treatment was a factor significantly influencing poorer OS (Table 5).

### 3.5. Complications of AML-pCT after Breast Cancer Treatment

The profile of treatment complications observed in AML-pCT patients following BC was comparable to that obtained in our previous report, which analyzed the general population of AML-pCT patients [13]. The most common toxic organ complications during intensive chemotherapy were hepatotoxicity (71.4%), renal toxicity (35.7%), and cardiotoxicity (21.4%). After alloHCT, renal toxicity (100.0%), hepatotoxicity (91.7%), and cardiotoxicity (25.0%) occurred most frequently (Table 6).

Within the majority of AML-pCT individuals with preceding BC, we have detected the presence of infectious complications during neutropenic episodes, and we evaluated them independently for patients treated with intensive chemotherapy and alloHCT. Within the alloHCT recipients, in the pre-engraftment period, fever of unknown origin (FUO) occurred in 100.0% of patients, and bacterial blood stream infections (BSI) occurred in 50.0%. In the post-engraftment period, viral infections were the most frequently observed (30.0%), followed by bacterial BSI in 20.0% of patients, and invasive fungal infections in 10.0% of the alloHCT recipients (Table 7).

After intensive chemotherapy, FUO was observed in 93.3% of individuals, while microbiologically documented infections, including BSI, were detected in 40.0% of patients.

Within AML-pCT after BC, in individuals undergoing alloHCT, GvHD occurred in three patients (acute GvHD in one patient and chronic GvHD in two patients).

Within AML-pCT patients with preceding BC that underwent intensive treatment, the most prevalent causes of death (9/21) were AML-pCT progression (44.4%), infections (22.2%), and toxicity (22.2%). After alloHCT, the main causes of death (4/12) comprised AML-pCT progression (*n* = 1), infections (*n* = 1), organ toxicity (*n* = 1), and acute GvHD (*n* = 1).

## 4. Discussion

In this paper, we focused on the extensive analysis of AML-pCT patients with preceding BC, one of the most common cancers worldwide. Both BC and AML-pCT genetic features, as well as treatment-related complications were taken into account, representing a valuable continuation of our previous studies on AML-pCT and a significant improvement compared to other reports.

Survival analysis using Kaplan–Meier curves revealed a higher OS in AML-pCT patients after BC treated with alloHCT and a lower OS in patients categorized into the adverse 2022 ELN genetic risk category and patients with CK. In multivariable and univariable Cox proportional hazard regression models, AML-pCT after BC treatment with alloHCT was the independent prognostic factor associated with a higher OS. Among patients intensively treated, the AML-pCT-MR post BC subtype was a factor associated with poorer survival in the univariate Cox proportional hazard regression model.

Over the past two decades, with BC being the most frequently identified prior malignancy, our finding revealed that AML-pCT after BC accounted for 35% of all AML-pCT cases. This stands in contrast to other studies, in which BC had a lower frequency and lymphomas were more commonly observed [16,17,18]. The total incidence of AML-pCT in our report (9.4%) was comparable to that in others [19,20]. In comparison to our previous report, we observed an increase in the general incidence of AML-pCT, particularly a notable increase in the occurrence of AML-pCT following BC, with 35.7% (10 out of 28) of such cases diagnosed in our center since August 2021 [13]. The incidence of myeloid neoplasms among BC survivors is underestimated and defined as 0.1–1.8% [15,21]. BC represents the most frequent female malignancy in Poland, based on reports between 2000 and 2023, with the increasing number of adjuvant therapy in the treatment of early-stage neoplasms possibly contributing to the growing number of cases [7].

Emerging findings indicate that the development of AML-pCT is influenced by complex factors, such as the acquisition of somatic mutations resulting from DNA-damaging cytotoxic therapy and inherited genetic cancer susceptibility [4]. Newer treatment methods, including poly (ADP-ribose) polymerase inhibitors (PARPi), were also associated with AML-pCT [4]. In a study, one in five AML-pCT patients after BC therapy was a carrier of germline mutations (most commonly in *BRCA1*, *TP53*, and *BRCA1* genes). Notably, those patients often had a family history of cancer within close relatives [5]. Given that AML-pCT following BC therapy continues to be a subtype associated with a poor prognosis, it is crucial for BC survivors with germline mutations to undergo close monitoring for the development of leukemia. In cases of bone marrow dysfunction, functional testing for these germline mutations should also be conducted [5].

Within the 2022 ELN AML diagnostic criteria, in the separate “AML with germline predisposition” category, it is highlighted that the *BRCA1* and *BRCA2* inherited mutations predispose not only to myeloid neoplasms but also to BC and ovarian cancers [14]. For individuals in the AML with germline predisposition patient group, comprising potential candidates for alloHCT from family donors, it is strongly advised to undergo genetic testing to identify germline risk alleles [14].

In our study group, we collected data concerning BC genetics only in six/28 of patients. In one patient, *PALB2* and *CHEK2* germline mutations were detected, and one patient was a carrier of the *CHEK2* germline mutation. The patient with *PALB2* and *CHEK2* mutations received intensive chemotherapy and alloHCT for the treatment of AML-pCT post BC. Moreover, the patient underwent prophylactic mastectomy and hysterectomy. The patient with the *CHEK2* mutation was diagnosed with APL-pCT. Germline mutations in *BRCA1*, *BRCA2*, *PALB2*, *TP53* and *CHEK2* genes can be observed in about 20% of acute leukemia patients with familial cancer predisposition syndromes [5]. We highly recommend verifying the BC genetic results to examine the presence of a germline mutation, especially in cases where family history suggests a predisposition to inherited cancers. There is a growing need for studies on inherited neoplastic susceptibility among AML-pCT patients with preceding BC.

The presence of clonal hematopoiesis of indeterminate potential (CHIP) prior to toxic exposure increased the risk of developing myeloid neoplasms post cytotoxic therapy by more than 13-fold [22]. Interestingly, among BC survivors treated with cytotoxic therapy who presented to breast oncologists with cytopenias and were subsequently referred for hematological investigation, 30.1% were diagnosed with AML-pCT (including AML-pCT-MR) [23]. An increased risk of secondary AML was observed among Caucasian patients compared to other races [24].

The median latency time in AML-pCT after BC observed in our report (5.0 years) is in line with previous reports [24,25]. We did not observe significant differences in the median latency time between patients with different prior cytotoxic treatment methods. The exact doses of cytotoxic therapy for BC were collected only in a limited number of patients in our study. Moreover, while the data concerning the impact of BC treatment with radiotherapy on the occurrence of AML-pCT are unclear, more recent data have questioned the increased risk for myeloid neoplasms [15,26]. It should be noted that brachytherapy or intensity-modulated radiotherapy can help reduce the risk of AML-pCT by optimizing the radiation dose [27]. The type of prior cytotoxic therapy did not influence the OS in our report; however, the study group was relatively small.

Among AML-pCT patients previously affected by BC, we found a higher frequency of *FLT3-ITD* (17.6%) and *NPM1* mutations (14.3%) but a lower frequency of *TP53* mutations (8.3%) compared to a genetic study on BC and gynecological cancer survivors who developed AML-pCT [28]. While the frequency of CK (19.0%) was lower in our report, the incidence of cytogenetic abnormalities was higher than previously reported (90.5%) [28]. Altogether, *TP53* mutation or 17p13 deletion were found in 28.6% of AML-pCT individuals previously affected by BC. Interestingly, in our previous study, we reported a presence of a novel c.989T>C *TP53* DNA sequence variant according to the COSMIC database, detected in one AML-pCT after BC patient from our hematological center included in the study group [13,29]. Moreover, in our study, among patients undergoing intensive treatment, the AML-pCT-MR following the BC subtype of disease was a factor associated with poorer survival, which may be explained by the acquisition of novel driver variants and the presence of molecular features associated with the leukemic progression of prior myelodysplastic neoplasms [30]. Among the AML-pCT-MR subgroup with preceding BC, we noted a higher frequency of adverse risk categories (80.0%) (2022 ELN) and a higher frequency of the *FLT3-ITD* mutation (33.3%) compared to AML-pCT after BC.

Our publication confirms the favorable outcome of APL-pCT following BC in one patient, despite the presence of the *CHEK2* germline mutation (detected at BC diagnosis) and an unfavorable AML-pCT genetic abnormality, involving the deletion of chromosome 17p13. This aligns with our previous report on the general AML-pCT population, which showed favorable outcomes within the APL-pCT subgroup [13].

Within our study period (2000–2023), the AML diagnostic strategy evolved, with molecular biology and cytogenetic examinations being performed in our center since 2008. The precise number of molecular biology and cytogenetic abnormalities might have only been revealed by performing an analysis of retrospectively collected samples.

The vast majority of AML-pCT with preceding BC were classified into the adverse 2022 ELN risk group category (54.2%) and, importantly, differences in OS were observed between 2022 ELN categories in our Kaplan–Maier curves. With the absence of a dedicated stratification tool for AML-pCT with preceding BC, we recommend adhering the 2022 ELN criteria and strongly emphasize the importance of testing for molecular abnormalities.

The median time from BC diagnosis to alloHCT was longer in our study than in the EBMT registry study [31]. We proved that alloHCT significantly extended OS within AML-pCT patients after BC when compared to chemotherapy alone, as stated by previous research [32]. We reported comparable OS and PFS rates within AML-pCT patients following BC that received alloHCT to those in the EBMT registry study [31]. Abnormal karyotype and a lack of disease control at alloHCT had a negative impact on OS and PFS [31], while older age and conditioning regimen intensity did not show any impact on the outcome [31]. In our report, MAC was performed less frequently (25.0%) than in the other study (39.0%) [31]. Hence, alloHCT should be performed as soon as possible, preferably in CR in AML-pCT patients with preceding BC.

Comparable to our previous report based on the general AML-pCT population and the findings of others, AML-pCT progression was the major cause of death within AML-pCT patients with preceding BC [13,33]. The main causes of death among AML-pCT patients with preceding BC that received alloHCT were AML-pCT progression and GvHD infections. In our report, none of patients died due to BC recurrence, while, according to others, BC relapse was the cause of death in up to 7% of alloHCT recipients [31].

AML-pCT patients with preceding BC are at higher risk of severe treatment-related complications [9]. The most common organ toxicities observed among patients after intensive chemotherapy and alloHCT were hepatotoxicity, renal toxicity, and cardiotoxicity, supporting the findings of our previous report based on the general AML-pCT population [13].

Infectious complications were reported to a high extent both after intensive chemotherapy and alloHCT, representing the second cause of death in AML-pCT patients with preceding BC. After intensive chemotherapy, during neutropenia, we observed Gram-positive bacteria BSI predominance, while a predominance of Gram-negative bacteria was observed after alloHCT [13,34,35]. Hence, further progress in prophylaxis and treatment of infectious complications is crucial to improve the outcomes of AML-pCT following BC [13].

Certain limitations should be acknowledged in this study: (1) data concerning BC genetics, including germline predisposition, were collected only in a limited number of patients, (2) detailed information on the dose and type of cytotoxic therapy was registered in a small number of BC survivors, (3) a small number of patients underwent comprehensive molecular characterization, and (4) AML-pCT after BC therapeutic strategies evolved over the two decades of the study.

## 5. Conclusions

Our findings reveal that (1) AML-pCT after BC stratification using the 2022 ELN criteria demonstrates a high frequency of adverse genetic risk categories; (2) patients with AML-pCT with preceding BC, characterized by CK, or classified into the adverse genetic ELN 2022 category, and those intensively treated with myelodysplasia-related subtype of AML-pCT, exhibit poorer survival rates; (3) the presence of germline mutations should be taken into consideration in AML-pCT post BC patients; (4) similarly to our previous report on AML-pCT, we observed that (4a) the aim of the treatment strategy in AML-pCT after BC should be based on performing alloHCT at the soonest opportunity, and (4b) further progress in the management of infection- and toxicity-related complications remains necessary to improve AML-pCT after BC outcomes.

## Figures and Tables

**Table 1:** Cytotoxic therapy and genetics of breast cancer among AML-pCT with known BC medical history.

Lp.	Age at BC Diagnosis	BC: Germline Mutational Status	BC Chemotherapy (Details)	BC Radiotherapy (Details)	BC Surgery (Details)	BC Hormone Therapy	Latency Time (years)	AML Post MDS	Other Neoplasm (Details of Treatment)	AML Cytogenetics	AML Molecular Biology Results	AML Treatment	Months of OS (Death/ Alive)
1	54	not tested	yes (A + C, TAX)	yes	yes (mastectomy)	no	5	yes	no	trisomy 8	*FLT3-TKD* mutation	CTH, alloHCT	19 (alive)
2	44	no data	yes (A + C)	yes (52.5 Gy)	yes (BCS)	yes	8	no	no	46XX, del(7) (q22) [15]	not performed	CTH, alloHCT	133 (alive)
3	43	no data	yes	yes (50–60 Gy with cobalt)	yes (Halsted)	yes	37	yes	no	not performed	not performed	AZA	34 (alive)
4	46	no	yes (4× E + C, 11× TAX)	no	yes (mastectomy)	yes	2	no	no	46, XX, t (?6;11) (?q27;q23) [11]/46,XX [2]	t (9;11); *KMT2A::MLLT3*	CTH	3 (death)
5	48	no	no	yes (52.5 Gy)	yes (BCS)	yes	7	no	second breast cancer (BCS and radiotherapy)	metaphases not analyzable	not performed	CTH, alloHCT	18 (alive)
6	38	yes; positive: *PALB2* c.509_510delGA p. (Arg170fs), *CHEK2* c.470T > C p. (Ile157Thr), (negative: *BRCA1*, *BRCA2*, *TP53*)	yes (1× A + C, 3× E + C)	no	yes (mastectomy + prophylactic mastectomy and hysterectomy)	yes	5	no	no	45, X, −X, t(8;21) (q22;q22) [9]/46, XX, t(8;21) (q22;q22) [2]	*C-KIT* mutation, t (8;21); *RUNX1::RUNX1T1*	CTH, alloHCT	24 (alive)
7	37	no	yes (4× A + C)	yes (56 Gy)	yes (BCS)	yes	2	no	no	inv(16); *CBFB::MYH11*	deletion of chromosome 17p13	CTH, alloHCT	171 (alive)
8	41	no data	yes (4× A + C, 4× TAC)	yes	yes (mastectomy)		5	no	no	metaphases not analyzable	deletion of chromosome 17p13	CTH, alloHCT	9 (death)
9	53	no	no	yes (52.5 Gy)	yes (BCS)	yes	5	no	no	47, XX, +8 [12]/46,XX [1]	*FLT3-ITD* mutation	CTH	5 (alive)
10	50	yes: positive: *CHEK2* c.470T > C p. (Ile157Thr), (negative: *BRCA1*, *BRCA2*)	yes (5× A + C, 4× TAC)	yes (50 Gy)	yes (mastectomy)	yes	5	yes	no	46, XX, t(15;17) (q24;q21) [16]/46, XX, del (7) (q36), t(15;17) (q24;q21) [3]/46, XX [1]	t (15;17); *PML::RARA*	All-trans retinoic acid, arsenic trioxide with CTH	81 (alive)
11	55	not tested	yes (6× A + C)	no	yes (mastectomy)	no	7	no	no	45, X, −X, t (8;21) (q22;q22) [17]/46, XX, t(8;21) (q22;q22) [1]	t (8;21); *RUNX1::RUNX1T1*	CTH	104 (alive)
12	60	no data	yes (4× A + C, 12× TAX)	yes (50 Gy)	yes (BCS, SNB)	no	3	yes	no	complex karyotype	t (2;11); *KMT2A* rearranged	CTH	20 (death)
13	49	not analyzed	yes	yes	yes (mastectomy)	no	20	no	uterine cancer (surgery, radiotherapy-brachytherapy, chemotherapy), malignant melanoma (surgery)	50~52, XX, +X [3], +1 [3], +6 [3], +9 [3], +10 [3], +11 [3], −13 [3], +21 [2], +mar1 [1], +mar2 [2], +mar3 [1] [cp3]/46, XX [5]	not performed	AZA + VEN	5 (alive)
14	71	no data	no	yes (40 Gy)	yes (BCS)	yes	3	no	no	metaphases not analyzable	not performed	AZA	11 (death)

Abbreviations: A, adriamycin; AZA, azacitidine; BC, breast cancer; BCS, breast conserving surgery; C, cyclophosphamide; CTH, intensive chemotherapy; E, epirubicine; SNB, sentinel node biopsy; OS, overall survival; TAC, docetaxel; TAX, paclitaxel; VEN, venetoclax.

**Table 2:** Clinical features of AML-pCT after breast cancer.

Patients’ Characteristics	AML-pCT after Breast Cancer (*n* = 28)
Age at AML-pCT diagnosis, years	57.5 (50.5–64.5)
Age at breast cancer diagnosis, years	49.0 (43.0–55.0)
Latency time, years	5.0 (4.0–7.0)
Race: white	28
Primary cytotoxic therapy:	
Chemotherapy	7
Radiotherapy	9
Both	12
Laboratory parameters:	
WBC, G/L	2.3 (1.2–16.4)
NEU, G/L	0.8 (0.2–2.5)
HGB, mmol/L,	5.6 (0.9)
PLT, G/L	86.0 (29.3–146.8)
BM blasts, %	41.0 (26.0–80.0)
AML-pCT post BC subtypes	
APL-pCT post BC	1
AML-pCT-MR post BC	6

Variables are noted as mean (standard deviation), median (interquartile range), or number of patients unless indicated otherwise. Abbreviations: WBC, white blood cell count; NEU, neutrophil count; HGB, hemoglobin; PLT, platelet count; BM, bone marrow; APL-pCT, acute promyelocytic leukemia post cytotoxic therapy; AML-pCT-MR, acute myeloid leukemia post cytotoxic therapy, myelodysplasia-related.

**Table 3:** Cytogenetic and molecular characteristics of AML-pCT after breast cancer.

Cytogenetic or Molecular Marker	*n/N*%
Cytogenetic assessment	
Metaphases not analyzable	4/25 (16.0)
Metaphases analyzable	21/25 (84.0)
Normal karyotype	2/21 (9.5)
Cytogenetic abnormalities	19/21 (90.5)
Deletion of chromosome 17p13	5/21 (23.8)
Complex karyotype ^a^	4/21 (19.0)
Deletion of chromosome 5	3/21 (14.3)
Monosomal karyotype ᵇ	2/21 (9.5)
t (8;21); *RUNX1::RUNX1T1*	2/21 (9.5)
Deletion of chromosome 7	2/21 (9.5)
t (15;17); *PML::RARA*	1/21 (4.8)
t (v;11q23.3); *KMT2A* rearranged	1/21 (4.8)
inv (16) or t (16;16); *CBFB::MYH11*	1/21 (4.8)
DNA sequence variants	
*FLT3—ITD* (cDNA)	3/17 (17.6)
*NPM1*	2/14 (14.3)
*TP53*	1/12 (8.3)
*FLT3—TKD (D835)* (cDNA)	1/17 (5.9)
*C-KIT*	1/1
ELN 2022 genetic risk stratification [14]	
Adverse	13/24 (54.2)
Intermediate	8/24 (33.3)
Favorable	3/24 (12.5)

^a^ Defined by ELN2022 as ≥3 chromosome abnormalities in the absence of other class-defining recurring genetic abnormalities; excludes hyperdiploid karyotype with three or more trisomies (or polysomies) without structural abnormalities; ᵇ defined as ≥2 autosomal monosomies or 1 autosomal monosomy and 1 structural abnormality; abbreviations: C- KIT, KIT proto-oncogene, receptor tyrosine kinase; n, number of positive results; N, number of tested patients; NPM1, nucleophosmin 1; ELN, European Leukemia Network [14].

**Table 4:** Treatment of AML-pCT after breast cancer.

Treatment Characteristics	AML-pCT after Breast Cancer (*n* = 28)
Type of treatment:	
Palliative care	3
Non-intensive therapy	5
Azacitidine	2
Azacitidine with venetoclax	3
Intensive treatment	20
Intensive chemotherapy	19
All-trans retinoic acid, arsenic trioxide with chemotherapy	1
AlloHCT	12
Targeted therapy with midostaurin	2
Treatment with 1st induction:	
Cytarabine and daunorubicin	16
Cytarabine, daunorubicin and cladribine	3
Complete remission	13
Without complete remission	5
Incomplete hematologic recovery	1
NEU recovery (0.5 G/L), days	28.1 (19.4)
No NEU recovery, *n*	1
PLT recovery (>50 G/L), days	25.9 (17.7)
No PLT recovery, *n*	4
AlloHCT	
Identical sibling	1
Matched unrelated donor	11
NEU recovery (0.5 G/L), days -	16.91 (3.51)
PLT recovery (>50 G/L), days -	18.0 (14.0–24.0)
AlloHCT in CR1	9
AlloHCT in CR2, CR3 or active disease	3
Stem cell source: peripheral blood	12
Time from breast cancer diagnosis to alloHCT, years	5.8 (5.2–7.4)
Time from AML-pCT diagnosis to alloHCT, months	9.5 (8.0–11.0)
Myeloablative conditioning	3
Reduced-intensity conditioning	9

Variables are noted as mean (standard deviation), median (interquartile range), or number of patients unless indicated otherwise. Abbreviations: NEU, neutrophil count; PLT, platelet count; alloHCT, allogenic hematopoietic cell transplant; CR, complete remission.

**Table 5:** Cox proportional hazards regression model to predict overall survival in AML-pCT after breast cancer.

Factor	Univariate		Multivariate	
	HR [95%, CI]	*p* Value	AHR [95%, CI]	*p* Value
Latency time, years	0.90 [0.74–1.09]	0.29		
Age at t-AML diagnosis				
≥65 years old	2.01 [0.58–6.92]	0.27		
<65 years old	1 (ref)			
t-AML treatment				
Palliative	1 (ref)		1 (ref)	
Intensive chemotherapy	0.35 [0.08–1.53]	0.16	0.37 [0.07–1.74]	0.21
AlloHCT	0.12 [0.03–0.56]	0.007	0.07 [0.01–0.80]	0.033
Non-intensive treatment	0.15 [0.02–1.49]	0.11	0.0 [0.00–10.14]	0.98
Type of previous cytotoxic therapy				
Radiotherapy	1 (ref)			
Chemotherapy	1.10 [0.25–4.93]	0.90		
Radiotherapy + chemotherapy	1.57 [0.43–5.76]	050		
Laboratory parameters at diagnosis				
BM blasts, %	1.00 [0.98–1.02]	0.82		
WBC, G/L	1.00 [0.99–1.01]	0.82		
NEU, G/L	0.98 [0.86–1.11]	0.73		
HGB, mmol/L	0.51 [0.24–1.10]	0.09		
PLT, G/L	1.00 [0.99–1.00]	0.47		
2022 ELN genetic risk category [14]				
Favorable	0.00 [9.06–1.72]	0.96	0.0 [1.61–79.90]	0.96
Intermediate	1 (ref)		1 (ref)	
Adverse	7.19 [0.91–56.48]	0.06	1.01 [0.08–13.59]	0.99
Cytogenetic abnormalities				
Complex karyotype	3.33 [0.93–11.92]	0.06	2.99 [0.67–13.48]	0.15
Non-complex karyotype	1 (ref)		1 (ref)	
Treatment with first induction:				
Complete remission	0.64 [0.17–2.39]	0.51		
Without complete remission	1 (ref)			
AML-pCT post BC subtype:				
AML-pCT	1 (ref)			
AML-pCT-MR	1.82 [0.55–6.03]	0.33		
AML-pCT post BC intensively treated:				
AML-pCT	1 (ref)			
AML-pCT-MR	5.95 [1.30–27.33]	0.022		

Abbreviations: AHR, HR adjusted for AML-pCT treatment, complex karyotype, ELN 2022 risk subgroup; BM, bone marrow; CI, confidence interval; ELN, European Leukemia Network; HGB, hemoglobin level; HR, hazard ratio; NEU, neutrophil count; PLT, platelet count; WBC, white blood cells count.

**Table 6:** Organ complications and adverse events during treatment of AML-pCT post breast cancer.

AlloHCT (*n* = 12)		Intensive Chemotherapy (*n* = 20)	
Complication	*n*, %	Complication	*n*, %
Renal toxicity ^a^ (total)	12 (100.0)	Hepatotoxicity ^a^	10 (71.4)
0–30 days after alloHCT	7 (58.3)	grade 1/grade 2/grade 3/grade 4	5/3/1/1
grade 1/grade 2/grade 3	3/3/1	Renal toxicity ^a^	5 (35.7)
30–100 days after alloHCT	10 (90.1)	grade 1/grade 2/grade 3	5/0/0
grade 1/grade 2/grade 3	5/4/1	Cardiotoxicity ^a^	3 (21.4)
Hepatotoxicity ^a^ (total)	11 (91.7)	grade 1/grade 2/grade 3	3/0/0
0–30 days after alloHCT	9 (75.0)	Neurotoxicity	4 (28.6)
grade 1/grade 2/grade 3	4/3/2	Deep vein thrombosis	2 (14.3)
30–100 days after alloHCT	9 (81.8)	Psychiatric	2 (14.3)
grade 1/grade 2/grade 3	8/0/1	Iatrogenic adverse events	1 (7.1)
Cardiotoxicity ^a^	3 (25.0)		
grade 1/grade 2/grade 3	1/0/0		
Hemorrhagic cystitis	1 (8.3)		
Pulmonary fibrosis	1 (8.3)	No data	6 (30.0)

^a^ According to the Common Terminology Criteria for Adverse Events (version 5.0), renal toxicity is defined as an increase in serum creatinine levels, hepatotoxicity is defined as an increase in alanine aminotransferase and alkaline phosphatase levels, and cardiotoxicity is defined as an increase in cardiac troponin I levels.

**Table 7:** Infectious complications in AML-pCT after breast cancer.

Type of Infections	AlloHCT (*n* = 12)			Intensive Chemotherapy (*n* = 20)
	≤30 Days	>30 Days	Total	
Fever of unknown origin	10 (100.0)	2 (20.0)	10 (100.0)	14 (93.3)
Bacterial blood stream infections	5 (50.0)	2 (20.0)	6 (60.0)	6 (40.0)
Gram-negative	4 (40.0)	1 (10.0)	4 (40.0)	3 (20.0)
*Escherichia coli*	2 (20.0)	-	2 (20.0)	1 (6.7)
*Klebsiella pneumoniae*	1 (10.0)	1 (10.0)	1 (10.0)	1 (6.7)
*Stenotrophomonas maltophilia*	-	-	-	1 (6.7)
*Serratia marcescens*	1 (10.0)		1 (10.0)	-
Gram-positive	1 (10.0)	1 (10.0)	2 (20.0)	5 (33.3)
*Staphylococcus aureus*	-	-	-	2 (13.3)
*Staphylococcus epidermidis*	-	-	-	1 (6.7)
*Enterococcus faecium*	1 (10.0)	1 (10.0)	2 (20.0)	1 (6.7)
*Staphylococcus haemolyticus*	-	-	-	1 (6.7)
Viral infections	-	3 (30.0)	3 (30.0)	-
*Cytomegalovirus* (CMV)	-	3 (30.0)	3 (30.0)	-
Fungal infections	-	-	-	-
Serum galactomannan	-	1 (10.0)	1 (10.0)	-
Data unavailable	2 (20.0)	2 (20.0)	2 (20.0)	5 (26.3)

Data are shown as number (percentage) of patients.

## Data Availability

The dataset is available from the corresponding author upon reasonable request.
